# Predictive factors associated with the persistence of chest pain in post-laparoscopic myotomy and fundoplication in patients with achalasia

**DOI:** 10.3389/fmed.2022.941581

**Published:** 2022-10-14

**Authors:** Héctor Olvera-Prado, José Peralta-Figueroa, Sofía Narváez-Chávez, Mario E. Rendón-Macías, Andric Perez-Ortiz, Janette Furuzawa-Carballeda, Silvia Méndez-Flores, María del Carmen Núñez-Pompa, Alonso Trigos-Díaz, Rodrigo Areán-Sanz, Fidel López-Verdugo, Enrique Coss-Adame, Miguel A. Valdovinos, Gonzalo Torres-Villalobos

**Affiliations:** ^1^Department of Surgery, Instituto Nacional de Ciencias Médicas y Nutrición Salvador Zubirán, Mexico City, Mexico; ^2^Escuela de Medicina, Universidad Panamericana, Mexico City, Mexico; ^3^Department of Immunology and Rheumatology, Instituto Nacional de Ciencias Médicas y Nutrición Salvador Zubirán, Mexico City, Mexico; ^4^Department of Dermatology, Instituto Nacional de Ciencias Médicas y Nutrición Salvador Zubirán, Mexico City, Mexico; ^5^Department of Gastroenterology, Instituto Nacional de Ciencias Médicas y Nutrición Salvador Zubirán, Mexico City, Mexico; ^6^Department of Experimental Surgery, Instituto Nacional de Ciencias Médicas y Nutrición Salvador Zubirán, Mexico City, Mexico

**Keywords:** achalasia, chest pain, dysphagia, predictive factors, post-laparoscopic myotomy

## Abstract

**Background:**

Episodic angina-like retrosternal pain is a prevalent symptom for achalasia patients pre- and post-treatment. The cause of postoperative chest pain remains poorly understood. Moreover, there are no reports on their predictive value for chest pain in the long-term post-treatment. The effect of laparoscopic Heller myotomy (LHM) and fundoplication techniques (Dor vs. Toupet) is unclear.

**Methods:**

We analyzed a cohort of 129 achalasia cases treated with LHM and randomly assigned fundoplication technique. All the patients were diagnosed with achalasia by high-resolution manometry (HRM). Patients were followed up at 1-, 6-, 12-, and 24-month post-treatment. We implemented unadjusted and adjusted logistic regression analyses to evaluate the predictive significance of pre- and post-operative clinical factors.

**Results:**

Preoperative chest pain with every meal was associated with an increased risk of occasional postoperative chest pain [unadjusted model: odds ratio (OR) = 12, 95% CI: 2.2–63.9, *P* = 0.006; adjusted model: *OR* = 26, 95% *CI*: 2.6–259.1, *P* = 0.005]. In type II achalasia, hypercontraction was also associated with an increased risk of chest pain (unadjusted model: *OR* = 2.6 e^9^ in all the patients). No significant differences were associated with age, type of achalasia, dysphagia, esophageal shape, and integrated relaxation pressure (IRP) with an increased risk of occasional postoperative chest pain. Also, there was no significant difference between fundoplication techniques or surgical approaches (e.g., length of myotomy).

**Conclusion:**

Preoperative chest pain with every meal was associated with a higher risk of occasionally postoperative chest pain.

## Introduction

Achalasia is a rare primary esophageal motor disorder characterized by the absence of swallow-induced relaxation of the lower esophageal sphincter and aperistalsis along the esophageal body (1–5). Although the etiology remains unclear, an interaction between autoimmune and inflammatory responses in genetically susceptible individuals leads to the loss of inhibitory neurons (and their mediators) in the myenteric esophageal plexus (4–10). This neuronal injury produces food transit impairment, which clinically manifests as dysphagia, regurgitation of saliva or undigested food, weight loss (1–4, 11), and episodic angina-like retrosternal pain (12–20). Of these symptoms, chest pain markedly lowers the quality of life and might overshadow typical symptoms leading to delayed diagnosis (16, 18). In addition, it is the most often underrecognized symptom by surgeons in the postoperative period (20).

Chest pain occurs in approximately 36–66% of pre- and 11–77.3% of post-achalasia treatment in different cohorts (12–20). Most pharmacological treatments are ineffective against this symptom, partly because the onset mechanism is poorly understood (16). Several pathways conducive to chest pain have been proposed. However, no single cause has been directly linked to this symptom. Currently, high-amplitude repetitive contractions stimulating esophageal mechanoreceptors (12, 13, 15, 16), the direct chemical stimulation of receptors on the esophageal mucosa (16, 21), the improved esophageal emptying post-myotomy (17, 22, 23), and its association with time since diagnosis (12) or gastroesophageal reflux disease are plausible factors under research (18, 24). Moreover, its remission in the post-treatment period by laparoscopic Heller myotomy (LHM), pneumatic dilation (PD), or Botox administration is still under evaluation (12–20).

Prior reports have indirectly assessed some of these mechanisms in prospective studies (12–20). However, the significance of clinical factors (e.g., age, time since diagnosis, HRM, and pHmetry findings) as surrogates of these pathophysiology pathways are contradictory and merit replication. Furthermore, no study has systematically assessed the probability of multiple occurrences of this symptom during the follow-up. Instead, all the studies have focused on describing clinical characteristics associated with patients affected by chest pain or evaluated a short-period post-treatment (12–20). To date, the relationship between patient characteristics [age, sex, body mass index (BMI), and time with achalasia]; barium swallow (grade of esophageal dilation, and shape), and high-resolution manometry (HRM) findings (achalasia type, pressures, and contraction vigor), the effect of treatment (type of fundoplication, and longitude of myotomy) and the change in HRM, pHmetry, and clinical scoring [Eckardt, DeMeester, Eating Assessment Tool-10 (EAT-10), Gastroesophageal Reflux Disease-Health Related Quality of Life (GERD-HQRL)] during follow-up on chest pain incidence are conflicting (12–20).

Since 2012, we have followed 129 achalasia patients who underwent LHM and randomly allocated fundoplication (Dor vs. Toupet). In this cohort, we monitored changes in clinical characteristics (incidence of symptoms) and diagnostic study findings (HRM) for over 8 years. Here, with these long-term assessments, we aimed to estimate the cumulative incidence of chest pain, accounting for multiple episodes during follow-up, at 48 months post-LHM. Moreover, assess the effect of preclinical and postoperative characteristics (type of fundoplication, longitude of myotomy, symptom questionnaires, barium swallow, and HRM findings) on long-term chest pain.

## Materials and methods

### Study design

A cohort study was followed by a randomized controlled trial to compare Dor vs. Toupet fundoplication after LHM. We analyzed 129 achalasia cases. A full description of this cohort is available elsewhere (25). All the cases were evaluated and followed-up at Instituto Nacional de Ciencias Médicas y Nutrición, Salvador Zubirán in Mexico City from 2012 to 2018 under an Institutional Review Board (IRB) approved protocol (IRB#: 1522). This study attained the Declaration of Helsinki principles, and written informed consent was obtained from all the subjects.

### Population

All the recruited cases had a confirmed diagnosis of achalasia by barium esophagogram, upper endoscopy, and HRM. We analyzed adult patients (≥ 18 years or older) without prior history of Chagas disease, esophageal stricture, gastric or esophageal cancer, peptic stricture, other esophageal motility disorders, or previous surgical treatment. At baseline, we performed detailed interviews and clinical assessments to collect demographic data and clinical history of achalasia. Achalasia symptoms were tracked at baseline and follow-up with the following international standardized, validated questionnaires: (1) Eckardt for dysphagia, regurgitation, chest pain, and weight loss (26); (2) GERD-HRQL to evaluate GERD symptoms (27); and finally (3) EAT-10 for dysphagia (28). For chest pain and dysphagia frequency pre- and postoperatively, we implemented the following scale: (1) any symptom, (2) occasional or intermittent, (3) daily, or (4) experiencing symptom with every meal.

### Interventions and follow-up

Before surgery, all the patients underwent an upper endoscopy, barium swallow, and HRM before and after surgery, and an upper gastrointestinal series and upper endoscopy were performed on all the patients. Postoperative assessments included upper endoscopy, pHmetry, HRM, and clinical evaluation of symptoms (chest pain and dysphagia frequency, Eckardt, GERD-HRQL, and EAT-10) conducted at 1-, 6-, 12-, 24-, and 48-month postoperatively.

### High-resolution manometry and 24-h pH monitoring

High-resolution manometry assessments were performed with a 360 high-resolution catheter with 36 channels (Medtronic ManoScan™, Minneapolis, MN, USA). We considered vigorous achalasia with any distal amplitude contractions greater than 37 (29–31). A 24-h pH monitoring was performed by a Digitrapper™ (Medtronic Minneapolis, MN, USA) pH-Z with a Versaflex catheter. We considered pathological finding any DeMeester score higher than 14.7 and any abnormal pH higher than 1.6% of reflux (32).

### Statistical approach

Descriptive statistic was performed, and categorical variables were compared using the chi-squared test or Fisher’s exact test. Continuous variables including age, BMI, disease evolution, questionnaires, neutrophil to lymphocyte ratio (NLR), pneumatic dilation sessions, esophageal dilation, basal lower esophageal sphincter (LES) pressure, IRP, and distal contractile integral analysis were performed using the Kruskal–Wallis one-way ANOVA on ranks. All the pairwise multiple comparison procedures were done by Dunn or Holm–Sidak method. Correlations among age, BMI, disease evolution, questionnaires, NLR, basal LES pressure, IRP, and distal contractile integral analysis were done using the Pearson correlation coefficient. Statistical analyses were performed using the Sigma Stat 14.5 program (Aspire Software International, Leesburg, VA, USA). Data are expressed as the median, range, and mean ± SD/SEM. *P*-values less than or equal to 0.05 were considered significant.

We then plotted the incidence of chest pain frequency (none, occasional, daily, and every meal) at the different evaluations as shown in Figures 2A,B and compared these symptoms to dysphagia frequency (using the same time frame) as shown in Figure 2B. To determine the differences between the presurgical score and 1-month post-surgery, McNemar–Bowker’s test was performed. Due to the losses of the patients, the results at 6-, 12-, and 24-months post-surgery only are shown as a descriptive analysis. For dysphagia, only pre-surgery vs. 1-month post-surgery collapsing to 4 boxes for expected values less than 1. To estimate the predictive value of pre- and postoperative patient characteristics and account for multiple chest pain reports during follow-up, we implemented a binary logistic regression model including non-adjusted and adjusted (age, type of achalasia, dysphagia, esophageal shape, and IRP were adjusted) models using IBM SPSS version 24.0 program (33).

**FIGURE 1:**
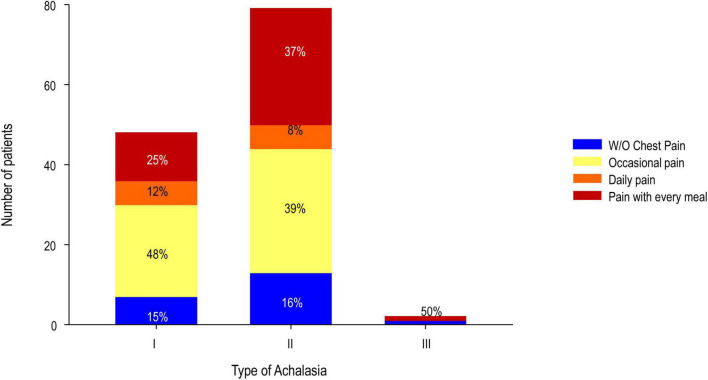
Presurgical chest pain according to the type of achalasia.

**FIGURE 2:**
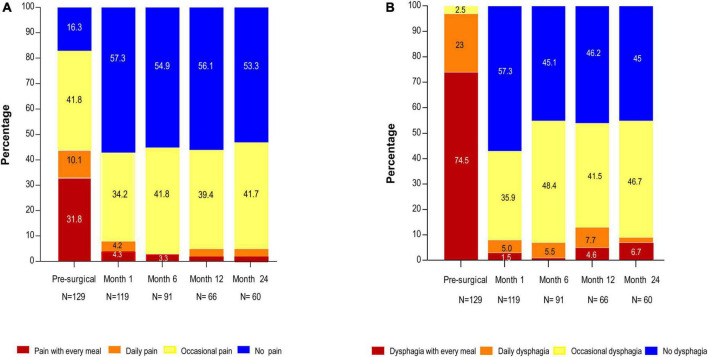
**(A)** Chest pain and **(B)** dysphagia at presurgery, 1-, 6-, 12-, and 24-month post-surgery.

## Results

### Patient characteristics and long-term chest pain incidence

As we previously reported, in a 2-year follow-up, the outcome of LHM with partial Dor or Toupet fundoplications had comparable improvement in symptom scores and HRM parameters. It was very effective and safe (34). A detailed description of our cohort at presurgery is available in Table 1. Of 129 evaluated cases, type II achalasia (61%) was the most prevalent, followed by type I achalasia (37%; Figure 1). Chest pain (84%) was the second most common symptom presurgically behind dysphagia (100%; Figures 2A,B). When the group was divided into patients without (*n* = 21) and with chest pain (occasional pain, daily pain, and pain with every meal; *n* = 108), it was found that patients with pain had an Eckardt score (9.2 ± 2.2 vs. 8.0 ± 4.5; *P* < 0.050), GERD-HRQL score (26.1 ± 12.3 vs. 13.8 ± 6.6; *P* < 0.050), and EAT score (31.1 ± 8.3 vs. 21.2 ± 8.3; *P* < 0.050) higher than patients without chest pain (Table 1).

**TABLE 1:** Presurgical characteristics of the study population according to chest pain.

	Total	W/o chest pain	With chest pain	*P*-value	*P*-value	*P*-value
	(*n* = 129)	(*n* = 21)	(*n* = 108)	Total vs. w/o CP	Total vs. w/CP	w/o vs. w/CP
**Demographics**						
Age (years), mean ± SD	40.6 ± 14.2	39.7 ± 13.7	40.8 ± 14.3	0.973		
median	39.0	38.0	39.5			
range	18–78	18–68	18–78			
Sex: female; n, (%)/male; n, (%)	74 (57)/55 (43)	10 (48)/11 (52)	64 (59)/44 (41)	NS	NS	NS
Ratio F/M	1.35	0.91	1.45			
**Clinical variables**						
BMI (Kg/m^2^), mean ± SD	23.2 ± 4.8	24.1 ± 5.7	23.0 ± 4.6	0.730		
median	22.5	23.1	22.4			
range	14.6–41.2	14.6–36.8	15.6–41.2			
Overweight; n, (%)	27 (21)	5 (24)	22 (20)	NS	NS	NS
Obesity; n, (%)	12 (9)	4 (19)	8 (7)	NS	NS	NS
Autoimmune disease; n, (%)	14 (11)	1 (5)	13 (12)	NS	NS	NS
Inflammatory diseases; n, (%)	22 (17)	1 (5)	21 (19)	NS	NS	NS
**Type of achalasia**						
I; n, (%)	48 (37)	7 (33)	41 (38)	NS	NS	NS
II; n, (%)	79 (61)	13 (62)	66 (61)			
III; n, (%)	2 (2)	1 (5)	1 (1)			
Disease evolution (Months), mean ± SD	25.5 ± 26.2	20.1 ± 25.9	26.5 ± 26.3	0.239		
median	14.5	10.0	16.0			
range	1–144	1–108	1–144			
**Symptoms**						
Chest pain; n, (%)	108 (84)	0 (0)	108 (100)	**<0.00001**	NS	**<0.00001**
Dysphagia; n, (%)	129 (100)	21 (100)	108 (100)	NS	NS	NS
Liquids; n, (%)	10 (8)	2 (10)	8 (7)	NS	NS	NS
Solids; n, (%)	38 (29)	8 (38)	30 (28)	NS	NS	NS
Both; n, (%)	81 (63)	11 (52)	70 (65)	NS	NS	NS
Regurgitation; n, (%)	120 (93)	19 (90)	100 (93)	NS	NS	NS
Weight loss, n, (%)	120 (93)	21 (100)	98 (91)	NS	NS	NS
**Questionnaires**						
Eckart (score), mean ± SD	9.0 ± 2.7	8.0 ± 4.5	9.2 ± 2.2	**<0.050**	NS	**<0.050**
median	9.0	8.0	9.0			
range	3–26	3–26	4–13			
GERD-HRQL (score), mean ± SD	24.1 ± 12.5	13.8 ± 6.6	26.1 ± 12.3	**<0.050**	NS	**<0.050**
median	22.0	12.0	23.5			
range	4–49	4–27	5–49			
EAT-10 (score), mean ± SD	29.4 ± 9.1	21.2 ± 8.3	31.1 ± 8.3	**<0.050**	NS	**<0.050**
median	32.0	22.0	34.0			
range	4–43	4–35	4–43			
**Viral exanthemata’s childhood disease**						
Chickenpox; n, (%)	91 (71)	14 (67)	77 (71)	NS	NS	NS
Measles; n, (%)	41 (32)	4 (19)	37 (34)	NS	NS	NS
Rubella; n, (%)	11 (9)	0 (0)	11 (10)	NS	NS	NS
Hepatitis; n, (%)	3 (2)	1 (5)	2 (2)	NS	NS	NS
Mumps; n, (%)	11 (9)	3 (14)	8 (7)	NS	NS	NS
**Environmental exposure**						
Tobacco exposure; n, (%)	40 (31)	9 (43)	31 (29)	NS	NS	NS
Biomass exposure; n, (%)	32 (25)	4 (19)	28 (26)	NS	NS	NS
**Laboratory data**						
Anti-nuclear antibodies; (%)	39/108 (36)	9/19 (47)	30/89 (34)			
Neutrophil to lymphocyte ratio mean ± SD	2.2 ± 1.2	2.1 ± 0.9	2.2 ± 1.3	0.980		
median	1.9	1.9	1.9			
range	0.6–7.7	1.1–4.8	0.6–7.7			
**Previous treatments**						
Pneumatic dilation; n, (%)	70 (54)	12 (57)	58 (54)	NS	NS	NS
Pneumatic dilation sessions, mean ± SD	1.3 ± 0.6	1.0 ± 0.1	1.4 ± 0.7	NS	NS	NS
median	1.0	1.0	1.0			
range	1–3	1–1	1–3			
**Preoperative diagnostic studies**						
**Barium swallow**						
Esophageal dilatation (cm), mean ± SD	4.9 ± 1.7	4.1 ± 1.8	5.1 ± 1.6	0.066		
median	4.7	4.1	5.0			
range	0.0–10.5	0.0–8.2	0.0–10.5			
Esophageal shape, n (%)						
Flask	89 (69)	12 (57)	77 (71.3)	NS	NS	NS
Spindle	28 (22)	7 (33)	21 (19.3)	NS	NS	NS
Sigmoid	12 (9)	2 (10)	10 (9.3)	NS	NS	NS
Degree esophageal dilatation						
First degree^†^; n, (%)	23 (18)	6 (29)	17 (15)	NS	NS	NS
Second degree^†^; n, (%)	65 (50)	11 (52)	59 (55)	NS	NS	NS
Third degree^†^; n, (%)	33 (26)	2 (10)	32 (30)	NS	NS	NS
**High-resolution manometry**						
Basal LES pressure (mmHg), mean ± SD	41.1 ± 24.5	37.2 ± 18.1	41.9 ± 25.5	0.857		
median	38.7	35.8	38.8			
range	11.7–162.0	13.0–80.0	11.7–162.0			
IRP (mmHg), mean ± SD	29.4 ± 13.8	28.3 ± 15.2	29.6 ± 13.5	0.740		
median	28.0	24.1	28.3			
range	5.1–82.2	11.2–81.0	5.1–82.2			
IRP > 15 mmHg; n, (%)	121 (94)	19 (90)	102 (94)	NS	NS	NS
Amplitude distal contractions,						
mean ± SD	15.4 ± 23.2	22.2 ± 26.7	14.0 ± 22.3	0.396		
median	0.0	0.0	0.0			
range	0.0–104.8	0.0–71.0	0.0–104.8			
Distal contractile integral (mmHg/s/cm), mean ± SD	1072.9 ± 4340.0	538.8 ± 1193.9	1184.4 ± 4738.6	0.893		
median	0.0	0.0	0.0			
range	0.0–37970	0.0–4611.0	0.0–37970			
Ineffective (Fail or weak); n, (%)	82 (73)	17 (81)	65 (60)	NS	NS	NS
Failed peristalsis (DCI < 100); n, (%)	77 (68)	15 (71)	62 (57)	NS	NS	NS
Weak peristalsis (DCI > 100 but < 450); n, (%)	5 (4)	2 (10)	3 (3)	NS	NS	NS
Normal (DCI > 450 but < 8,000); n, (%)	20 (18)	4 (19)	16 (15)	NS	NS	NS
Hypercontractile (> 8,000); n, (%)	4 (3)	0 (0)	4 (4)	1	1	1
Vigorous achalasia (Amplitude distal contractions > 37); n, (%)	21 (19)	7 (33)	14 (13)	0.0743	0.582	**0.0458**

Numbers may not sum to totals due to missing data, and column percentages may not sum to 100% due to rounding. BMI, body mass index; cm, centimeter; DCI, distal contractile integral; HRM, High-resolution manometry; IRP, Integrated relaxation pressure; Kg, kilogram; LES, Lower esophageal sphincter; min, minute; mmHg, millimeters of mercury; NS, Not significant; ps, pre-surgery; s, second; SD, standard deviation; w/o, without, w/o CP, without chest pain; w/CP, with chest pain. ^†^ First degree of dilatation (< 3.5 cm), second degree (≥ 3.5 < 6 cm), third-degree (≥ 6). In bold statistically significant (*p* < 0.05) variables.

There was no difference between the length of the esophageal myotomy, gastric myotomy, and full myotomy (Supplementary Table 1). There was no difference in the number of patients with a Dor fundoplication (63%) vs. Toupet (47%).

**FIGURE 3:**
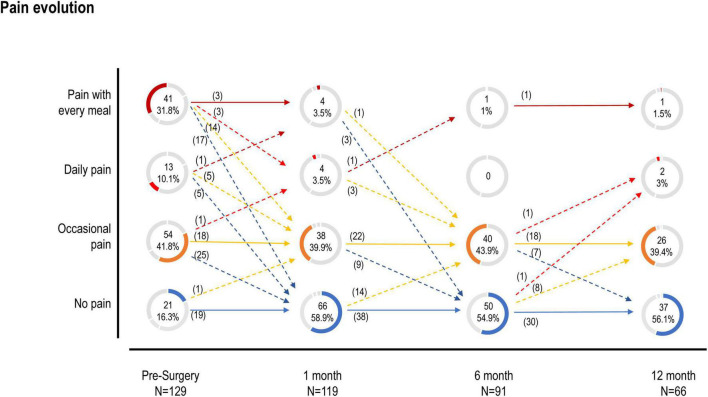
Pain evolution.

A 1 month after surgery, the number of patients without chest pain increased 3-fold (*n* = 69) compared to presurgery patients (*n* = 21; Figures 2A,B). Dysphagia decreased in patients without and with chest pain (25 and 64 vs. 100%; *P* < 0.00001; Figure 3 and Supplementary Table 1) compared to presurgery. Patients without chest pain had lower Eckardt score (1.2 ± 1.3 vs. 3.0 ± 2.3; *P* < 0.001; Supplementary Table 1), GERD-HRQL score (3.1 ± 4.5 vs. 5.9 ± 5.4 *P* = 0.001; Table 2), and EAT score (2.1 ± 1.3 vs. 5.6 ± 7.1; *P* = 0.004; Supplementary Table 1) than patients with chest pain.

**TABLE 2:** Non-adjusted logistic regression analysis of occasional chest pain vs. no pain at 12 months after surgery (*n* = 66).

Variables *Pre-surgery*		OR	[CI95%]	Wald	*P* value
**Sex**					
	Female	1.7	[0.72; 4.3]	1.57	0.20
	Male	1			
**Age**					
	<30	Ref			
	30–50	1.3	[0.5; 3.7]	0.38	0.53
	50	1.5	[0.5;4.8]	0.59	0.44
**Type achalasia**					
	I	1			
	II	0.7	[0.27; 1.6]		0.53
	III	0	[anybody had pain]		
**Dysphagia**					
	Occasional	1			
	Daily	1.74	[0.12; 23.9]	0.16	0.68
	With every meal	1.45	[0.12; 16.7]	0.08	0.76
**Esophageal shape**					
	Sigmoid	1			
	Flask	2.6	[0.49; 13.4]	1.25	0.26
	Spindle	3.9	[0.63; 23.8]	2.16	0.14
**Contraction vigor**					
	Normal	1			
	Hypercontraction	2.6 e^9^	[all *N* = 2]		
	Weaked	0.92	[0.06; 12.3]	0.004	0.94
	Failed	1.18	[0.36; 3.6]	0.08	0.76
IRP*					
	Normal	1			
	<15	0.67	[0.17; 2.4]	0.42	0.51
**Chest pain**					
	No pain	1			
	Occasional	6.7	[1.3; 33.2]	5.25	**0.02**
	Daily	6.4	[0.8; 45.9]	3.4	**0.06**
	With every meal	12	[2.2; 63.9]	8.46	**0.006**

IRP, Integrated relaxation pressure. In bold statistically significant (*p* < 0.05) variables.

A 6 months after surgery, the patients without chest pain (*n* = 50) had lower Eckardt score (1.1 ± 1.5 vs. 2.8 ± 1.8; *P* < 0.001; Table 3), EAT score (1.3 ± 2.2 vs. 5.6 ± 7.2; *P* = 0.002; Supplementary Table 2), and DeMeester score > 14.72 [21.8 ± 4.3 (*n* = 7) vs. 45.5 ± 29.6 (*n* = 6); *P* = 0.044; Supplementary Table 2] than patients with chest pain (*n* = 41).

**TABLE 3:** Adjusted logistic regression analysis of occasional chest pain vs. no pain at 12 months after surgery associated with presurgical chest pain (*n* = 66).

Chest pain					
	No pain	1			
	Occasional	12.4	[1.3; 116.4]	4.38	**0.028**
	Daily	5.2	[0.4; 72.9]	1.51	**0.22**
	With every meal	26.1	[2.6; 259.1]	7.78	**0.005**

Adjusted with sex, age, dysphagia, esophageal shape, contraction vigor and, Integrated relaxation pressure. In bold statistically significant.

A 12 months after surgery, the patients without chest pain (*n* = 37) had lower Eckardt score (0.8 ± 1.3 vs. 2.3 ± 1.7; *P* < 0.001; Supplementary Table 3) and disease evolution (24.6 ± 22.6 vs. 25.9 ± 16.0 months; *P* = 0.004; Supplementary Table 3) than patients with chest pain (*n* = 29). The number of patients with dysphagia and without chest pain was also lower (38 vs. 66%; *P* = 0.0464; Figure 3 and Supplementary Table 3) compared with patients with dysphagia and chest pain.

A 24 months after surgery, the patients without chest pain (*n* = 32) remained with a sustained reduction in the score of the questionnaires Eckardt score (0.9 ± 1.2 vs. 3.0 ± 1.8; *P* < 0.001; Supplementary Table 4), GERD-HRQL score (4.5 ± 6.9 vs. 7.2 ± 4.3; *P* = 0.003; Supplementary Table 4), and EAT score (2.6 ± 4.9 vs. 5.1 ± 4.7; *P* = 0.011; Supplementary Table 4) compared with patients with chest pain (*n* = 28).

The chest pain with every meal decreased at 1-, 6-, 12-, and 24-month post-surgery compared with presurgery (4.3, 3.3, 1.5, 1.7 vs. 31.8%; *P* < 0.0001; Figure 2A). The daily chest pain also decreased at 1-, 6-, 12-, and 24-month post-surgery vs. presurgery (4.3, 0, 3.0, 3.3 vs. 10.1%; Figure 2A). Conversely, the percentage of patients who did not have chest pain increased at 1-, 6-, 12-, and 24-month post-surgery vs. presurgery (57.3, 54.9, 56.1, 53.3 vs. 16.3%; *P* < 0.0001; Figure 2A; McNemar–Bowker: presurgery vs. 1 month *X*^2^ = 61.8^6*fd*^ *P* < 0.0001).

The evolution of chest pain in patients with achalasia during the first year is shown in Figure 3.

Regarding dysphagia with every meal and daily dysphagia diminished at 1-, 6-, 12-, and 24-month post-surgery compared with presurgery (Figure 1B, McNemar: presurgery vs. 1 month with every meal or daily vs. occasional or no pain X^2^ 99.1_1*fd*_ *P* = 001). Dysphagia disappeared at 1-, 6-, 12-, and 24-month post-surgery in 57.3, 45.1, 46.2, and 45.0% of patients, while all the patients had dysphagia before surgery (Figure 2B).

### Correlation between chest pain and Eckardt score

We found positive correlation between the chest pain and Eckardt score (Pearson’s *r* = 0.641, *P* = 1.741e^–13^ at presurgery; *r* = 0.656, *P* = 3.264e^–6^ at 6 months post-surgery; *r* = 0.521, *P* = 3.740e^–3^ at 12 months post-surgery; and *r* = 0.565, *P* = 1.720e^–3^ at 24 months post-surgery). We did not find significant correlations between GERD-HRQL and EAT-10 scores, age, BMI, disease evolution, NLR, basal LES pressure, IRP, and distal contractile integral.

### Predictive value of clinical characteristics

The pre- and postoperative patient characteristics with significant predictive value were analyzed in a binary logistic regression model, including non-adjusted and adjusted (age, type of achalasia, dysphagia, esophageal shape, and IRP) models at 12 months post-surgery. Results are shown in Tables 2, 3, respectively.

Preoperative chest pain with every meal was associated with an increased risk of occasional postoperative chest pain [unadjusted model: odds ratio (*OR)* = 12, 95% *CI*: 2.2–63.9, *P* = 0.006; adjusted model: *OR* = 26, 95% *CI*: 2.6–259.1; *P* = 0.005].

Preoperative episodic angina-like retrosternal pain daily was associated with an increased risk of occasional postoperative chest pain (unadjusted model: *OR* = 6.4, 95% *CI*: 0.8–45.9, *P* = 0.06; adjusted model: *OR* = 5.2, 95% *CI*: 0.4–72.9, *P* = 0.22).

Preoperative occasional chest pain was associated with an increased risk of occasional postoperative chest pain (unadjusted model: *OR* = 6.7, 95% *CI*: 1.3–33.2, *P* = 0.02; adjusted model: *OR* = 12.4, 95% *CI*: 1.3–116.4, *P* = 0.028).

In type II achalasia, hypercontraction was also associated with an increased risk of chest pain (unadjusted model: *OR* = 2.6 e^9^ in all the patients). No significant differences were associated with age, type of achalasia, dysphagia, esophageal shape, and IRP with an increased risk of occasional postoperative chest pain. Also, there was no significant difference between fundoplication techniques or surgical approaches (e.g., length of myotomy).

A multiple linear regression analysis at 24 months was performed to examine the influence of age, disease evolution (months), BMI, GERD-HRQL, GERD pyrosis, EAT-10, Eckardt score, dysphagia, basal LES pressure, IRP, amplitude distal contractions, and distal contractile integral (DCI) on the variable chest pain. The regression model showed that the variables explained 89.32% of the variance from the chest pain variable. An ANOVA was used to test whether this value differed significantly from zero. The present sample showed that the effect was significantly different from zero, *F* = 8.36, *P* ≤ 0.001, *R*^2^ = 0.89 (Supplementary Tables 5–7).

## Discussion

This study evaluated the long-term incidence of chest pain after a laparoscopic myotomy and fundoplication for achalasia. We built upon prior works identifying critical clinical factors associated with this symptom, pre- and post-surgical. However, we implemented a comprehensive approach by analyzing the risk for multiple symptom recurrence up to 24 months post-LHM. By this approach, preoperative pain frequency positively predicted postoperative episodic angina-like retrosternal pain persistence. We also found a positive correlation between chest pain and Eckardt score in each time evaluated. Notwithstanding our comprehensive approach, in our study, other clinical features with a significant association in prior works (12–20) were not predictive factors. There was no significant association between other patients (age, sex, achalasia features, HRM, and pHmetry findings), and postoperative (fundoplication techniques or surgical approach, serial upper endoscopies, and HRMs) characteristics with chest pain.

The cause and mechanism of episodic angina-like retrosternal pain in achalasia or other esophageal motility disorders remain poorly understood (16). Several clinical reports support that high amplitude, repetitive contractions stimulating esophageal mechanoreceptors might be a critical pathogenesis pathway (12, 13, 15, 16). However, there have been some challenges to this theory – first, a weak correlation between chest pain prevalence and the degree of esophageal motor abnormalities and function. For instance, identical LES pressures and esophageal body diameters have been documented between cases with chest pain and those without chest pain (12). Second, the lack of association between pain episodes and altered esophageal contraction amplitudes (12). Last, the persistence of this symptom despite the disappearance of abnormal contractions and/or dysphagia (12). Our study supports this mechanism since a hypercontractile esophagus significantly increased the chest pain risk by 2.6 e^9^. This predictive factor was independent of age and sex. Especially, age independence is crucial since several reports describe an age-dependent relation to chest pain incidence (12, 14). It has also been suggested that as the disease progresses and esophageal dilatation develops, esophageal contractions decrease amplitude, and chest pain eventually subsides in most patients (15). We did not observe that dysphagia increased the risk of chest pain.

Laparoscopic Heller myotomy significantly benefits dysphagia with a response rate of approximately 90% (19, 25, 34–37). Moreover, chest pain improvement has also been reported (15, 21, 38, 39).

Like other cohorts, chest pain prevalence preoperatively was 81%, and post-surgical fluctuated between 17 and 76%. However, the latter was experienced occasionally. Our results agree with prior research demonstrating that LHM immediately impacts the short postoperative period, especially for daily and every meal symptoms (16). However, we evidenced that this benefit is significant for the latter. By the 48th month, three out of four patients will intermittently complain about this symptom. Nevertheless, postoperatively, dysphagia in the long term was occasionally experienced, arguing for reasonable control. The significance of complete remission in patient pain reporting warrants future studies.

One limitation of our study is that we did not follow-up our cohort with a timed barium esophagogram. This could provide valuable insights into the effect of esophageal emptying that need future dissection. However, we preferred implementing pHmetry and HRM assessments over timed barium esophagograms for completeness in information in this matter. Moreover, at the 48th month of follow-up, we had only information on 28% (33/118) of individuals. Hence, our incidence estimations may be biased in this stratum. Nevertheless, our study contributes to long-term assessments evidencing trends described above, not previously reported.

## Data availability statement

The raw data supporting the conclusions of this article will be made available by the authors, without undue reservation.

## Ethics statement

The studies involving human participants were reviewed and approved by the Ethics and Research Committee of the Salvador Zubirán National Institute of Medical Sciences and Nutrition. The patients/participants provided their written informed consent to participate in this study.

## Author contributions

HO-P, JP-F, SN-C, AP-O, and MR-M performed the analysis and interpretation of the data, responsible for the study conception and design, and wrote the manuscript. HO-P, JP-F, SN-C, JF-C, MN-P, AT-D, RA-S, FL-V, EC-A, and MV collected patient’s data from the clinical files, created the database, analyzed and interpreted the data, and wrote the manuscript. GT-V analyzed the data, wrote the manuscript, critically revised the manuscript for important intellectual content, and was responsible for the study conception and design. All authors contributed to the article and approved the submitted version.
