# Supplementary material for: Predictive factors associated with the persistence of chest pain in post-laparoscopic myotomy and fundoplication in patients with achalasia
Source: Front Med (Lausanne). 2022 Oct 14;9:941581. doi: 10.3389/fmed.2022.941581 (PMC9614071; doi:10.3389/fmed.2022.941581)
Supplement: Supplementary file 1 [file Table_1.pdf]

Supplementary Table 1. Characteristics of the study population according to chest pain at one-month post-surgery.

	Total (n=119)	W/o Chest Pain (n=69)	With Chest Pain (n=50)	*P*-value Total vs. w/o CP	*P*-value Total vs. w/CP	*P*-value w/o vs. w/CP
**Demographics**						
Age (years), mean ± SD	40.7 ± 14.6^{ps}	41.5 ± 14.6^{ps}	39.5 ± 14.6^{ps}	0.710		
median	39.0	40.0	38.0			
range	18 – 78	18 – 70	18 – 78			
Sex: female; n, (%)/male; n, (%)	70 (59) / 49 (41)	39 (56) / 30 (43)	31 (62) / 19 (38)	NS	NS	NS
Ratio F/M	1.43	1.30	1.63			
**Clinical variables**						
BMI (Kg/m^2), mean ± SD	23.7 ± 4.8	23.6 ± 4.9	23.9 ± 4.6	0.950		
median	23.1	23.5	22.2			
range	15.4 – 39.2	15.4 – 39.2	17.4 – 38.0			
Overweight; n, (%)	26 (22)^{ps}	16 (23)^{ps}	10 (20)^{ps}	NS	NS	NS
Obesity; n, (%)	10 (8)^{ps}	8 (12)^{ps}	2 (4)^{ps}	NS	NS	NS
Autoimmune disease; n, (%)	14 (12)^{ps}	6 (9)^{ps}	8 (16)^{ps}	NS	NS	NS
Inflammatory diseases; n, (%)	20 (17)^{ps}	10 (15)^{ps}	10 (20)^{ps}	NS	NS	NS
**Type of Achalasia**						
I; n, (%)	41 (34)	23 (33)	18 (36)	NS	NS	NS
II; n, (%)	76 (64)	44 (64)	32 (64)	NS	NS	NS
III; n, (%)	2 (2)	2 (3)	0 (0)	NS	NS	NS
**Disease evolution (Months)**						
mean ± SD	26.0 ± 26.8^{ps}	25.5 ± 27.0^{ps}	26.7 ± 26.9^{ps}	1.0		
median	16.0	14.0	18.0			
range	1 – 144	1 – 144	2 – 115			
**Symptoms**						
Chest pain; n, (%)	50 (42)	0 (0)	50 (100)	<.00001	NS	<0.00001
Dysphagia; n, (%)	49 (41)	17 (25)	32 (64)	0.0265	0.0074	<0.00001
Regurgitation; n, (%)	23 (19)	9 (13)	14 (28)	NS	NS	NS
**Questionnaires**						
Eckart (score), mean ± SD	1.9 ± 2.0	1.2 ± 1.3	3.0 ± 2.3	0.007	0.001	<0.001
median	1.5	1.0	3.0			
range	0 – 12	0 – 6	0 – 12			
GERD-HRQL (score), mean ± SD	4.3 ± 5.1	3.1 ± 4.5	5.9 ± 5.4	NS	NS	0.001
	3.0	2.0	4.5			
median	0 - 24	0 - 22	0 - 24			

range						
EAT-10 (score), mean ± SD	3.6 ± 5.6	2.1 ± 1.3	5.6 ± 7.1	NS	NS	0.004
median	2.0	1.0	3.0			
range	0 - 35	0 - 6	0 - 35			
**Viral exanthemata's childhood disease**						
Chickenpox; n, (%)	83 (70)[ps]	49 (71)[ps]	34 (68)	NS	NS	NS
Measles; n, (%)	39 (33)[ps]	26 (38)[ps]	13 (26)	NS	NS	NS
Rubella; n, (%)	11 (9)[ps]	7 (10)[ps]	4 (8)	NS	NS	NS
Hepatitis; n, (%)	3 (3)[ps]	3 (4)[ps]	0 (0)	NS	NS	NS
Mumps; n, (%)	11 (9)[ps]	8 (12)[ps]	3 (6)	NS	NS	NS
**Environmental exposure**						
Tobacco exposure; n, (%)	37 (31)[ps]	23 (33)[ps]	14 (28)	NS	NS	NS
Biomass exposure; n, (%)	30 (25)[ps]	12 (17)[ps]	18 (36)	NS	NS	0.0316
**Laboratory Data**						
Anti-nuclear antibodies; (%)	37/103 (36)[ps]	20/59 (34)[ps]	17/44 (39)	NS	NS	NS
Neutrophil to lymphocyte ratio mean±SD	2.2 ±1.3[ps]	2.0 ±1.1[ps]	2.4 ±1.5[ps]	0.244		
median	1.9	1.8	2.0			
range	0.6 – 7.7	0.6 – 5.2	1.0 – 7.7			
**Previous Treatments**						
Pneumatic dilation; n, (%)	66 (56)[ps]	40 (58)[ps]	16 (32)	NS	NS	NS
Pneumatic dilation sessions,	1.3 ± 0.7[ps]	1.3 ± 0.7[ps]	1.3 ± 0.5[ps]	NS	NS	NS
mean ± SD	1.0	1.0	1.0			
median	1 – 3	1 – 3	1 – 2			
range						
**Diagnostic studies**						
**Barium swallow**						
Esophageal dilatation (cm),	4.8 ± 1.6[ps]	4.8 ± 1.7[ps]	4.6 ± 1.5[ps]	0.843		
mean ± SD	4.6	4.6	4.8			
median	0.0 - 10.5	0.0 - 10.5	0.0 – 7.5			
range						
Esophageal shape, n (%)						
Flask	81 (68)[ps]	47 (68)[ps]	34 (68)[ps]	NS	NS	NS
Spindle	27 (23)[ps]	15 (22)[ps]	12 (24)[ps]	NS	NS	NS
Sigmoid	11 (9)[ps]	7 (10)[ps]	4 (8)[ps]	NS	NS	NS
Degree esophageal dilatation						
First degree[†]; n, (%)	23 (19)[ps]	12(17)[ps]	11(22)[ps]	NS	NS	NS
Second degree[†]; n, (%)	59 (50)[ps]	33 (48)[ps]	26 (52)[ps]	NS	NS	NS
Third degree[†]; n, (%)	29 (24)[ps]	18 (26)[ps]	11 (22)[ps]	NS	NS	NS
**High-Resolution Manometry**						

Basal LES pressure (mmHg), mean ± SD<br>median<br>range	11.7 ± 10.1<br>10.4<br>0.0 – 77.0	11.1 ± 8.3<br>10.1<br>0.0 – 51.2	12.5 ± 12.2<br>11.7<br>0.1 – 77.0	0.934		
Integrated relaxation pressure (IRP) (mmHg), mean ± SD<br>median<br>range	7.9 ± 5.0<br>7.7<br>0.0 – 33.2	8.2 ± 5.7<br>7.8<br>0.0 – 33.2	7.5 ± 4.0<br>7.4<br>1.5 – 17.0	0.992		
IRP > 15 mmHg; n, (%)	6 (5)	5 (7)	1 (2)	NS	NS	NS
Amplitude distal contractions, mean ± SD<br>median<br>range	11.7 ± 20.7<br>0.0<br>0.0 – 126	11.8 ± 18.6<br>0.0<br>0 – 84.1	11.6 ± 23.8<br>0.0<br>0.0 – 126.0	0.860		
Distal contractile integral (mmHg/s/cm), mean ± SD<br>median<br>range	141.6 ± 470.1<br>0.0<br>0.0 – 3528.0	158.7 ± 404.3<br>0.0<br>0.0 – 2809.0	116.6 ± 557.8<br>0.0<br>0.0 – 3528.0	0.112		
**Surgical data**						
**Fundoplication technique**						
Dor; n, (%)	69 (63)	36 (52)	33 (66)	NS	NS	NS
Toupet; n, (%)	56 (47)	33 (48)	23 (46)			
**Intraoperative data**						
Esophageal myotomy (cm), mean ± SD<br>median<br>range	6.0 ± 0.7<br>6.0<br>4.0 -7.0	6.0 ± 0.7<br>6.0<br>4.0 -7.0	6.0 ± 0.7<br>6.0<br>4.0 -7.0	NS	NS	NS
Gastric myotomy (cm), mean ± SD<br>median<br>range	3.2 ± 0.5<br>3.0<br>3.0 - 4.0	3.2 ± 0.6<br>3.0<br>3.0 - 4.0	3.1 ± 0.3<br>3.0<br>3.0 - 4.0			
Full myotomy length (cm), mean ± SD<br>median<br>range	9.2 ± 0.8<br>9.0<br>7.0 – 12.0	9.1 ± 0.8<br>9.0<br>7.0 – 12.0	9.1 ± 0.7<br>9.0<br>7.0 – 11.0	NS	NS	NS
Dor Fundoplication length (cm), mean±SD<br>median<br>range	4.5 ± 0.6<br>4.0<br>3.0 – 6.0	4.5 ± 0.6<br>4.0<br>3.0 – 6.0	4.5 ± 0.6<br>4.5<br>3.0 – 6.0			
Toupet Fundoplication length (cm), mean ± SD	4.2 ± 0.5<br>4.0	4.2 ± 0.5<br>4.0	4.2 ± 0.5<br>4.0	NS	NS	NS

median range	3.0 – 6.0	3.0 – 6.0	3.0 – 5.0

Numbers may not sum to totals due to missing data, and column percentages may not sum to 100% due to rounding.

BMI: body mass index; cm: centimeter; DCI: distal; contractile integral; HRM: High-resolution manometry; IRP: Integrated relaxation pressure; Kg: kilogram; LES: Lower; esophageal sphincter; min: minute; mmHg: millimeters of mercury; NS: Not significant; ps: pre-surgery; s: second; SD: standard deviation; w/o: without, w/o CP: without chest pain; w/CP: with chest pain. †: First degree of dilatation (< 3.5 cm), second degree (≥ 3.5 < 6 cm), third-degree (≥ 6).

Supplementary Table 2. Characteristics of the study population according to chest pain at six-month post-surgery.

	Total (n=91)	W/O Chest Pain (n=50)	With Chest Pain (n=41)	*P*-value Total vs. w/o CP	*P*-value Total vs. w/CP	*P*-value w/o vs. w/CP
**Demographics**						
Age (years), mean ± SD	40.7 ±	39.0 ±	43.0 ±	0.551		
median	14.6[ps]	14.4[ps]	15.1[ps]			
range	39.0	39.0	40.0			
	18 – 78	18 – 68	19 – 78			
Sex: female; n, (%)/male; n, (%)	59 (65) / 32 (35)	29 (58) / 21 (42)	30 (73) / 11 (27)			
Ratio F/M	1.84	1.38	2.72			
**Clinical variables**						
BMI (Kg/m^2), mean ± SD	25.5 ± 4.8	25.8 ± 5.4	25.3 ± 4.1	0.997		
median	25.3	24.6	25.5			
range	17.6 – 43.5	19.0 – 43.5	17.6 – 34.5			
Overweight; n, (%)	20 (22)	9 (18)	11 (27)	NS	NS	NS
Obesity; n, (%)	8 (9)	5 (10)	3 (7)	NS	NS	NS
Autoimmune disease; n, (%)	7 (8)[ps]	2 (4)[ps]	5 (12)[ps]	NS	NS	NS
Inflammatory diseases; n, (%)	16 (18)[ps]	6 (12)[ps]	10 (24)[ps]	NS	NS	NS
**Type of Achalasia**						
I; n, (%)	23 (25)	11 (22)	12 (29)	NS	NS	NS
II; n, (%)	66 (73)	37 (74)	29 (71)	NS	NS	NS
III; n, (%)	2 (2)	2 (4)	0 (0)	NS	NS	NS
**Disease evolution (Months)**						
mean ± SD	24.7 ± 24.1	23.9 ±	25.7 ± 23.7	0.806		
median	[ps]	24.6[ps]	[ps]			
range	15.5	13.5	17.0			
	1 – 108	1 – 108	2 – 91			
**Symptoms**						
Chest pain; n, (%)	41 (45)	0 (0)	41 (100)	<0.00001	<0.00001	<0.00001
Dysphagia; n, (%)	50 (55)	23 (46)	27 (66)	0.3789	0.2587	0.0897
**Questionnaires**						
Eckart (score), mean ± SD	1.8 ± 1.8	1.1 ± 1.5	2.8 ± 1.8	<0.050	<0.050	<0.001
median	1.0	1.0	2.0			
range	0 – 9	0 – 8	0 – 9			
GERD-HRQL (score), mean ± SD	5.0 ± 6.0	3.3 ± 3.1	7.1 ± 7.9	0.071		
median	3.0	2.5	4.0			
range	0.0 – 33.0	0.0 – 13.0	0.0 – 33.0			
EAT-10 (score), mean ± SD	3.2 ± 5.5	1.3 ± 2.2	5.6 ± 7.2	NS	NS	0.002
median	1.0	1.0	4.0			

range	0.0 – 36.0	0.0 – 11.0	0.0 – 36.0			
**Viral exanthemata's childhood disease**						
Chickenpox; n, (%)	69 (76)[ps]	39 (78)[ps]	30 (73)[ps]	NS	NS	NS
Measles; n, (%)	33 (36)[ps]	16 (32)[ps]	17 (41)[ps]	NS	NS	NS
Rubella; n, (%)	8 (9)[ps]	3 (6)[ps]	5 (12)[ps]	NS	NS	NS
Hepatitis; n, (%)	2 (2)[ps]	1 (2)[ps]	1 (2)[ps]	NS	NS	NS
Mumps; n, (%)	10 (1)[ps]	7 (14)[ps]	3 (7)[ps]	NS	NS	NS
**Environmental exposure**						
Tobacco exposure; n, (%)	30 (33)[ps]	21 (42)[ps]	11 (27)[ps]	NS	NS	NS
Biomass exposure; n, (%)	23 (25)[ps]	12 (24)[ps]	11 (27)[ps]	NS	NS	NS
**Laboratory Data**						
Anti-nuclear antibodies; (%)	31/83 (37)[ps]	19/43 (44)[ps]	12/39 (31)[ps]	NS	NS	NS
Neutrophil to lymphocyte ratio	2.3 ±1.3[ps]	2.2 ±1.4[ps]	2.3 ±1.3[ps]	0.995		
mean±SD	2.0	2.0	2.0			
median	0.6 – 7.7	0.6 – 7.7	0.6 – 7.1			
range						
**Previous Treatments**						
Pneumatic dilation; n, (%)	56 (62)[ps]	29 (58)[ps]	27 (67)[ps]	NS	NS	NS
Pneumatic dilation sessions,	1.2 ± 0.5[ps]	1.3 ± 0.6[ps]	1.0 ± 0.0[ps]	NS	NS	NS
mean ± SD	1.0	1.0	1.0			
median	1 – 3	1 – 3	1 – 1			
range						
**Diagnostic studies**						
**Barium swallow**						
Esophageal dilatation (cm), mean	4.6 ± 1.6[ps]	4.8 ± 1.6[ps]	4.3 ± 1.5[ps]	0.707		
± SD	4.5	4.4	4.6			
median	0.0 - 10.5	2.0 - 10.5	0.0 – 6.5			
range						
Esophageal shape, n (%)						
Flask	61 (67)[ps]	34 (68)[ps]	27 (66)[ps]	NS	NS	NS
Spindle	19 (21)[ps]	9 (18)[ps]	10 (24)[ps]	NS	NS	NS
Sigmoid	11 (12)[ps]	7 (14)[ps]	4 (10)[ps]	NS	NS	NS
Degree esophageal dilatation						
First degree†; n, (%)	23 (25)[ps]	13 (26)[ps]	10 (24)	NS	NS	NS
Second degree†; n, (%)	48 (53)[ps]	27 (54)[ps]	21 (51)	NS	NS	NS
Third degree†; n, (%)	17 (19)[ps]	11 (22)[ps]	6 (15)	NS	NS	NS
**High-Resolution Manometry**						
Basal LES pressure (mmHg),	12.8 ± 7.7	14.8 ± 8.8	10.5 ± 5.3	0.123		
mean ± SD	11.3	13.0	11.1			
median	0 – 39.2	0.1 – 39.2	0.0 – 20.8			
range						
Integrated relaxation pressure (IRP) (mmHg), mean ± SD	7.9 ± 4.5	8.6 ± 4.9	7.0 ± 3.9	0.664		

median	7.8	7.6	7.8			
range	0.0 – 23.3	1.2 – 23.3	0.0 – 13.6			
IRP > 15 mmHg; n, (%)	4 (4)	4 (8)	0 (0)	NS	NS	NS
Amplitude distal contractions,						
mean ± SD	16.6 ± 32.7	19.0 ±	13.9 ± 27.3	0.931		
median	0.0	36.9	0.0			
range	0.0 – 201.5	0.0	0.0 – 130.7			
		0.0 – 201.0				
Distal contractile integral	235.3 ±	308.9 ±	152.0 ±	0.995		
(mmHg/s/cm), mean ± SD	923.5	1197.7	450.0			
median	0.0	0.0	0.0			
range	0.0 – 7750	0.0 – 7750	0.0 – 2188.7			
DeMeester score > 14.72; n,	13 (14)	7 (14)	6 (15)	NS	NS	NS
(%)						
DeMeester score > 14.72, mean	34.3 ± 24.6	21.8 ± 4.3	45.5 ± 29.6	NS	NS	0.044
± SD	23.7	21.7	34.1			
median	16.1 – 109.0	16.1 – 28.9	22.0 – 109.0			
range						

Numbers may not sum to totals due to missing data, and column percentages may not sum to 100% due to rounding.

BMI: body mass index; cm: centimeter; DCI: distal; contractile integral; HRM: High-resolution manometry; IRP: Integrated relaxation pressure; Kg: kilogram; LES: Lower; esophageal sphincter; min: minute; mmHg: millimeters of mercury; NS: Not significant; ps: pre-surgery; s: second; SD: standard deviation; w/o: without, w/o CP: without chest pain; w/CP: with chest pain. †: First degree of dilatation (< 3.5 cm), second degree (≥ 3.5 < 6 cm), third-degree (≥ 6).

Supplementary Table 3. Characteristics of the study population according to chest pain at twelve-months post-surgery.

	Total (n=66)	W/O Chest Pain (n=37)	With Chest Pain (n=29)	*P*-value Total vs. w/o CP	*P*-value Total vs. w/CP	*P*-value w/o vs. w/CP
**Demographics**						
Age (years), mean ± SD	40.7 ±	40.7 ±	42.4 ±	0.915		
median	14.6^ps	14.9^ps	14.9^ps			
range	39.0	40.0	41.0			
	18 – 78	18 – 68	19 – 78			
Sex: female; n,	37 (56) /	18 (49) /	19 (66) / 10			
(%)/male; n, (%)	29 (44)	19 (51)	(34)			
Ratio F/M	1.28	0.95	1.94			
**Clinical variables**						
BMI (Kg/m^2), mean ± SD	26.8 ± 6.1	28.0 ± 6.7	25.0 ± 4.8	0.349		
median	26.4	28.0	24.8			
range	19.0 – 46.8	19.0 – 46.8	20.2 – 35.5			
Overweight; n, (%)	10 (15)	7 (19)	3 10)	NS	NS	NS
Obesity; n, (%)	6 (9)	4 (11)	2 (7)	NS	NS	NS
Autoimmune disease; n, (%)	6 (9) ps	3 (8) ps	3 (10) ps	NS	NS	NS
Inflammatory diseases; n, (%)	6 (9) ps	2 (5) ps	4 (19) ps	NS	NS	NS
**Type of Achalasia**						
I; n, (%)	16 (24)	8 (22)	8 (28)	NS	NS	NS
II; n, (%)	48 (73)	27 (73)	21 (72)	NS	NS	NS
III; n, (%)	2 (3)	2 (5)	0 (0)	NS	NS	NS
**Disease evolution (Months)**	25.2 ±	24.6 ± 22.6	25.9 ± 16.0	NS	NS	0.004
mean ± SD	24.4 ps	ps	ps			
median	18.0	18.0	16.0			
range	1 – 108	2 – 108	1 – 96			
**Symptoms**						
Chest pain; n, (%)	29 (44)	0 (0)	29 (100)	<.000001	<0.00001	<0.00001
Dysphagia; n, (%)	36 (55)	14 (38)	19 (66)	0.15	0.3717	0.0464
**Questionnaires**						
Eckart (score), mean ± SD	1.5 ± 1.6	0.8 ± 1.3	2.3 ± 1.7	<0.050	<0.050	<0.001
median	1.0	1.0	2.0			
range	0 – 7	0 – 6	0 – 7			
GERD-HRQL (score), mean	4.8 ± 6.2	3.9 ± 5.2	6.0 ± 7.2	0.150		
± SD	3.0	2.0	4.0			
median	0.0 – 35.0	0.0 – 25.0	0.0 – 35.0			
range						

EAT-10 (score), mean ± SD	2.8 ± 4.9	2.2 ± 4.1	3.7 ± 5.6	0.072		
median	1.0	0.0	2.0			
range	0.0 – 29.0	0.0 – 21.0	0.0 – 29.0			
**Viral exanthemata's childhood disease**						
Chickenpox; n, (%)	52 (79)[ps]	31 (84)[ps]	21 (72)[ps]	NS	NS	NS
Measles; n, (%)	25 (38)[ps]	12 (38)[ps]	13 (45)[ps]	NS	NS	NS
Rubella; n, (%)	6 (9)[ps]	0 (0)[ps]	6 (21)[ps]	0.0853	0.1771	0.0052
Hepatitis; n, (%)	1 (2)[ps]	0 (0)[ps]	1 (3)[ps]	NS	NS	NS
Mumps; n, (%)	9 (14)[ps]	6 (16)[ps]	3 (10)[ps]	NS	NS	NS
**Environmental exposure**						
Tobacco exposure; n, (%)	22 (33)[ps]	14 (38)[ps]	8 (28)[ps]	NS	NS	NS
Biomass exposure; n, (%)	18 (27)[ps]	9 (27)[ps]	9 (31)[ps]	NS	NS	NS
**Laboratory Data**						
Anti-nuclear antibodies; (%)	25/61 (41)[ps]	15/34 (44)[ps]	10/26 (38)[ps]	NS	NS	NS
Neutrophil to lymphocyte						
ratio mean±SD	2.0 ± 0.9[ps]	2.0 ± 0.9[ps]	2.0 ± 0.9[ps]	0.975		
median	1.9	1.9	1.8			
range	0.6 – 4.8	0.6 – 4.8	1.1 – 4.5			
**Previous Treatments**						
Pneumatic dilation; n, (%)	44 (67)[ps]	26 (70)[ps]	18 (62)[ps]	NS	NS	NS
Pneumatic dilation	1.2 ± 0.6[ps]	1.3 ± 0.7[ps]	1.0 ± 0.0[ps]	NS	NS	NS
sessions, mean±SD	1.0	1.0	1.0			
median	1 – 3	1 – 3	1 – 1			
range						
**Diagnostic studies**						
**Barium swallow**						
Esophageal dilatation (cm),	4.4 ± 1.7[ps]	4.6 ± 1.8[ps]	4.2 ± 1.5[ps]	0.776		
mean ± SD	4.3	4.4	4.1			
median	0.0 - 10.5	0.0 - 10.5	0.0 – 7.5			
range						
Esophageal shape, n						
(%)	48 (73)[ps]	25 (67)[ps]	23 (79)[ps]	NS	NS	NS
Flask	10 (15)[ps]	5 (14)[ps]	5 (17)[ps]	NS	NS	NS
Spindle	8 (12)[ps]	7 (19)[ps]	1 (4)[ps]	NS	NS	NS
Sigmoid						
Degree esophageal dilatation						
First degree†; n, (%)	13 (21)[ps]	7 (21)[ps]	6 (21)	NS	NS	NS
Second degree†; n, (%)	39 (64)[ps]	21 (62)[ps]	18 (62)	NS	NS	NS
Third degree†; n, (%)	9 (15)[ps]	6 (18)[ps]	3 (10)	NS	NS	NS
**High-Resolution Manometry**						

Basal LES pressure (mmHg), mean ± SD<br>median<br>range	8.0 ± 5.4<br>5.8<br>2.8 – 17.1	9.8 ± 6.2<br>9.3<br>3.6 – 17.1	5.6 ± 3.7<br>4.2<br>2.8 – 9.8	0.606		
Integrated relaxation pressure (IRP) (mmHg), mean ± SD<br>median<br>range	5.2 ± 3.0<br>6.5<br>1.8 – 8.0	4.9 ± 3.5<br>4.8<br>1.8 – 8.0	5.7 ± 2.8<br>6.5<br>2.5 – 8.0	0.883		
IRP > 15 mmHg; n, (%)	0 (0)	0 (0)	0 (0)	NS	NS	NS
DeMeester score > 14.72; n, (%)	3 (5)	1 (3)	2 (7)	NS	NS	NS
DeMeester score > 14.72, mean ± SD<br>median<br>range	36.3 ± 25.0<br>29.1<br>15.8 – 64.1	15.8	46.6 ± 24.8<br>46.6<br>29.1 – 64.1	0.388		

Numbers may not sum to totals due to missing data, and column percentages may not sum to 100% due to rounding.

BMI: body mass index; cm: centimeter; DCI: distal; contractile integral; HRM: High-resolution manometry; IRP: Integrated relaxation pressure; Kg: kilogram; LES: Lower; esophageal sphincter; min: minute; mmHg: millimeters of mercury; NS: Not significant; ps: pre-surgery; s: second; SD: standard deviation; w/o: without, w/o CP: without chest pain; w/CP: with chest pain. †: First degree of dilatation (< 3.5 cm), second degree (≥ 3.5 < 6 cm), third-degree (≥ 6).

Supplementary Table 4. Characteristics of the study population according to chest pain at twenty-four months post-surgery.

	Total (n=60)	W/O Chest Pain (n=32)	With Chest Pain (n=28)	*P*-value Total vs. w/o CP	*P*-value Total vs. w/CP	*P*-value w/o vs. w/CP
**Demographics**						
Age (years), mean ± SD	42.4 ±	42.4 ±	42.3 ±	0.970		
median	14.6[ps]	14.0[ps]	15.4[ps]			
range	41.0	41.5	39.5			
	18 – 78	18 – 68	18 – 78			
Sex: female; n,	38 (63) /	20 (63) / 12	18 (64) / 10	NS	NS	NS
(%)/male; n, (%)	22 (37)	(37)	(36)			
Ratio F/M	1.73	1.67	1.80			
**Clinical variables**						
BMI (Kg/m^2), mean ± SD	27.3 ± 6.0	27.8 ± 6.6	26.7 ± 5.6	0.987		
median	26.0	26.0	26.3			
range	16.7 – 48.2	18.9 – 48.2	16.7 – 39.1			
Overweight; n, (%)	13 (22)	7 (22)	6 (21)	NS	NS	NS
Obesity; n, (%)	7 (11)	3 (9)	4 (14)	NS	NS	NS
Autoimmune disease; n, (%)	6 (10)[ps]	4 (12)[ps]	2 (7)[ps]	NS	NS	NS
Inflammatory diseases; n, (%)	12 (20)[ps]	7 (22)[ps]	5 (18)[ps]	NS	NS	NS
**Type of Achalasia**						
I; n, (%)	17 (28)	11 (35)	6 (21)	NS	NS	NS
II; n, (%)	41 (69)	19 (59)	22 (79)	NS	NS	NS
III; n, (%)	2 (3)	2 (6)	0 (0)	NS	NS	NS
**Disease evolution (Months)**						
mean ± SD	25.4 ±	24.1 ± 20.2	26.9 ± 28.6	0.959		
median	24.2[ps]	[ps]	[ps]			
range	15.0	18.0	13.0			
	2 – 108	3 – 82	2 – 108			
**Symptoms**						
Chest pain; n, (%)	28 (47)	0 (0)	28 (100)	<.000001	<.000001	<.000001
Dysphagia; n, (%)	28 (47)	11 (34)	17 (61)	NS	NS	NS
**Questionnaires**						
Eckart (score), mean ± SD	1.9 ± 1.8	0.9 ± 1.2	3.0 ± 1.8	0.002	<0.001	<0.001
median	1.5	1.0	3.0			
range	0 – 9	0 – 6	1 – 9			
GERD-HRQL (score), mean ±	5.8 ± 6.0	4.5 ± 6.9	7.2 ± 4.3	NS	NS	0.003
SD	4.0	2.0	6.0			
median	0.0 – 30.0	0.0 – 30.0	2.0 – 16.0			
range						
EAT-10 (score), mean ± SD	3.8 ± 5.0	2.6 ± 4.9	5.1 ± 4.7	NS	NS	0.011
median	1.5	1.0	3.0			

range	0.0 – 23.0	0.0 – 23.0	0.0 – 16.0			
**Viral exanthemata's childhood disease**						
Chickenpox; n, (%)	47 (78)[ps]	26 (81)[ps]	21 (75)[ps]	NS	NS	NS
Measles; n, (%)	26 (43)[ps]	12 (38)[ps]	14 (50)[ps]	NS	NS	NS
Rubella; n, (%)	7 (12)[ps]	2 (6)[ps]	5 (18)[ps]	NS	NS	NS
Hepatitis; n, (%)	1 (2)[ps]	0 (0)[ps]	1 (4)[ps]	NS	NS	NS
Mumps; n, (%)	8 (13)[ps]	6 (19)[ps]	2 (7)[ps]	NS	NS	NS
**Environmental exposure**						
Tobacco exposure; n, (%)	19 (32)[ps]	10 (31)[ps]	9 (32)[ps]	NS	NS	NS
Biomass exposure; n, (%)	16 (27)[ps]	9 (28)[ps]	7 (25)[ps]	NS	NS	NS
**Laboratory Data**						
Anti-nuclear antibodies; (%)	21/57 (37)[ps]	12/31 (39)[ps]	9/27 (33)[ps]	NS	NS	NS
Neutrophil to lymphocyte	2.3 ± 1.4[ps]	2.0 ± 0.9[ps]	2.7 ± 1.7[ps]	0.391		
ratio mean±SD	2.0	1.9	2.2			
median	0.6 – 7.7	0.6 – 4.8	1.1 – 7.7			
range						
**Previous Treatments**						
Pneumatic dilation; n, (%)	44 (73)[ps]	23 (72)[ps]	21 (75)[ps]	NS	NS	NS
Pneumatic dilation sessions,	1.2 ± 0.6[ps]	1.2 ± 0.7[ps]	1.3 ± 0.5[ps]	NS	NS	NS
mean ± SD	1.0	1.0	1.0			
median	1 – 3	1 – 3	1 – 2			
range						
**Diagnostic studies**						
**Barium swallow**						
Esophageal dilatation (cm),	4.5 ± 1.7[ps]	4.4 ± 1.9[ps]	4.5 ± 1.4[ps]	0.780		
mean ± SD	4.4	4.1	4.5			
median	0.0 - 10.5	0.0 - 10.5	0.0 – 7.5			
range						
Esophageal shape, n (%)						
Flask	42 (70)[ps]	20 (63)[ps]	22 (79)[ps]	NS	NS	NS
Spindle	12 (17)[ps]	8 (24)[ps]	4 (17)[ps]	NS	NS	NS
Sigmoid	6 (13)[ps]	4 (13)[ps]	2 (4)[ps]	NS	NS	NS
Degree esophageal dilatation						
First degree[†]; n, (%)	11 (21)[ps]	7 (26)[ps]	4 (14)	NS	NS	NS
Second degree[†]; n, (%)	33 (63)[ps]	16 (59)[ps]	17 (61)	NS	NS	NS
Third degree[†]; n, (%)	8 (15)[ps]	4 (15)[ps]	4 (14)	NS	NS	NS
**High-Resolution Manometry**						
Basal LES pressure (mmHg),	12.1 ± 6.1	13.5 ± 6.4	10.7 ± 5.5	0.968		
mean ± SD	10.8	13.3	9.8			
median	2.2 – 24.7	2.6 – 24.7	2.2 – 21.0			
range						

Integrated relaxation pressure (IRP) (mmHg), mean ± SD<br>median<br>range	7.6 ± 3.8<br>7.2<br>1.0 – 14.00	7.9 ± 3.6<br>8.4<br>1.0 – 13.6	7.4 ± 4.1<br>6.2<br>1.4 – 14.0	0.999			
IRP > 15 mmHg; n, (%)	0 (0)	0 (0)	0 (0)	NS	NS	NS	
Amplitude distal contractions, mean ± SD<br>median<br>range	9.7 ± 18.5<br>0.0<br>0.0 – 82.0	13.6 ± 22.3<br>0.0<br>0.0 – 82.0	5.7 ± 12.8<br>0.0<br>0.0 – 36.7	0.905			
Distal contractile integral (mmHg/s/cm), mean ± SD<br>median<br>range	128.4 ± 346.1<br>0.0<br>0 – 1993.0	190.8 ± 441.3<br>0.0<br>0.0 – 1993.0	63.3 ± 195.6<br>0.0<br>0.0 – 910	0.337			
DeMeester score > 14.72; n, (%)	11 (18)	6 (19)	5 (18)	NS	NS	NS	
DeMeester score > 14.72, mean ± SD<br>median<br>range	32.7 ± 17.0<br>28.2<br>15.5 – 68.6	37.8 ± 20.8<br>34.4<br>15.5 – 68.6	26.7 ± 9.8<br>23.5<br>18.0 – 42.9	0.571			

Numbers may not sum to totals due to missing data, and column percentages may not sum to 100% due to rounding.

BMI: body mass index; cm: centimeter; DCI: distal; contractile integral; HRM: High-resolution manometry; IRP: Integrated relaxation pressure; Kg: kilogram; LES: Lower; esophageal sphincter; min: minute; mmHg: millimeters of mercury; NS: Not significant; ps: pre-surgery; s: second; SD: standard deviation; w/o: without, w/o CP: without chest pain; w/CP: with chest pain. †: First degree of dilatation (< 3.5 cm), second degree (≥ 3.5 < 6 cm), third-degree (≥ 6).

Supplementary Table 5. A multiple linear regression analysis

R	R^2	Adjusted R^2	Standard error of the estimate
0.95	0.89	0.79	0.3

Supplementary Table 6. ANOVA

Model	df	F	p
Regression	12	8.36	<.001

Supplementary Table 7. Coefficients of the multiple linear regression analysis

	not standardized Coefficients	standardized Coefficients			
Model	B	Beta	Standard error	t	*p*
(Constant)	1.2		0.53	2.25	0.044
Aye (years)	-0.02	-0.41	0.01	-3.31	**0.006**
Disease evolution (months)	0.01	0.22	0	1.33	0.21
BMI (kg/m^2)	0	0.04	0.01	0.36	0.722
GERD-HRQL	0.12	0.9	0.05	2.48	**0.029**
GERD pyrosis	-0.18	-0.84	0.06	-2.95	**0.012**
EAT-10	-0.06	-0.43	0.03	-2.22	**0.046**
ECKARDT	0.52	1.6	0.09	5.66	**<0.001**
Dysphagia	-0.72	-0.95	0.2	-3.64	**0.003**
Basal LES pressure (mmHg)	-0.03	-0.22	0.02	-1.48	0.164
IRP (mmHg)	-0.01	-0.08	0.03	-0.46	0.652
Amplitude distal contractions	-0.01	-0.21	0.01	-0.72	0.483
DCI (mmHg/s/cm)	-0	-0.22	0	-0.83	0.425

BMI: body mass index; cm: centimeter; DCI: distal; contractile integral; HRM: High-resolution manometry; IRP: Integrated relaxation pressure; Kg: kilogram; LES: Lower; esophageal sphincter; min: minute; mmHg: millimeters of mercury; w/o: without, w/o CP: without chest pain; w/CP: with chest pain.
